# Diagnosis and Treatment Pathway of MDR/RR-TB in Taizhou, Zhejiang Province, China

**DOI:** 10.3390/tropicalmed8020079

**Published:** 2023-01-21

**Authors:** Jingting Lu, Yuanyuan Xu, Zhipeng Li, Xiaoxiao Chen, Haijiang Lin, Qi Zhao

**Affiliations:** 1 School of Public Health, Fudan University, Shanghai 200032, China; 2 Key Laboratory of Public Health Safety, Ministry of Education, Shanghai 200032, China; 3 Taizhou Center for Disease Control and Prevention, Zhejiang 318000, China; 4 Shanghai Municipal Center for Disease Control and Prevention, Shanghai 200336, China

**Keywords:** MDR/RR-TB, waiting time, attrition, treatment outcome, factors

## Abstract

This study aims to understand MDR/RR-TB patients’ experience from seeking TB-related health care to diagnosis and treatment completion, as well as the social determinants with the waiting time for DST and treatment, pre-treatment attrition, and treatment outcome based on a retrospective cohort study. Univariate and multi-variate logistic regressions were used to analyze the associated factors. The median time of waiting time for DST and treatment was 24.00 and 30.00 days, respectively. Non-residential patients (aOR: 2.89, 95% CI: 1.14–7.70), registered before 2018 (aOR: 19.93, 95% CI: 8.99–48.51), first visited a county-level hospital (aOR: 4.65, 95% CI: 1.08–21.67), sputum smear-negative (aOR: 3.54, 95% CI: 1.28–10.16), and comorbid with pneumoconiosis (aOR: 7.10, 95% CI: 1.23–47.98) had a longer DST delay. The pre-treatment attrition was 26.9% (82/305). Elderly, non-residential patients and patients registered before 2018 were more likely to refuse MDR/RR treatment. However, in housekeeping/unemployment and farmer/fisherman, recurrent patients tended to take therapeutic measures actively. The successful treatment rate was 62.1% (105/169). Elderly, comorbidity with diabetes and sputum smear conversion time >1 month may lead to poorer outcomes. Immediate interventions should be taken to smooth diagnosis and treatment pathways and improve the social protections further so as to encourage patients to cooperate with the treatment actively.

## 1. Introduction

Multidrug-resistant tuberculosis (MDR-TB), by having resistance to at least isoniazid and rifampicin, the two most effective first-line drugs [1], has become a global health problem along with rifampicin-resistant tuberculosis (RR-TB). Although it is a curable disease, the reported number of MDR/RR-TB patients enrolled in treatment remains small. Records show that only one-third of people who develop MDR/RR-TB each year initiate MDR/RR-TB treatment [1]; additionally, the treatment success rate from the latest patient cohort was 60% based on the currently available data [1]. Altogether, the MDR/RR-TB management still needs improvements, or else it could impede the implementation of the global “END TB Strategy”.

China is one of the 30 countries with a high TB burden, contributing to 13% of global MDR/RR-TB patients. The crucial next step should focus on expanding treatment coverage and increasing the treatment success rate [2,3] to advance TB elimination in China. Several identified challenges and factors resulting in poor treatment outcome [4] include delays in MDR/RR-TB diagnosis and treatment initiation, comorbidities, lower BMI, and so on [5]. The delays between these processes are a major factor [6,7,8]. A number of cases have revealed that there is often a prolonged waiting time for people awaiting drug susceptibility tests (DSTs) and for TB-diagnosed patients to receive effective treatment [9,10]. Chen Yong found that the median (IQR) waiting time for treatment initiation from reporting DST results was 51 (23–113) days in 2010–2014 in Shanghai, a city where medical facilities and policies are well established [9]. Several studies discussed the idea that delays in MDR/RR-TB diagnosis and treatment initiation may accelerate disease progression and lead to an increased probability of poor treatment outcomes for individuals or a higher risk of disease transmission, which can subsequently cause a catastrophic socioeconomic cost at the community level [8,11] and hinder the accomplishment of the “END TB Strategy”. Therefore, reducing the waiting time in the process of MDR/RR-TB diagnosis and treatment is a crucial precondition in future MDR/RR-TB control in China.

The Chinese government attaches great importance to the management and treatment of tuberculosis (TB) [12,13]. Growing applications in new technology, such as GeneXpert, and social protections have been invested in to close the waiting time gap in the diagnosis and treatment pathway (DTP) of MDR/RR-TB [13,14]. Currently, effective MDR/RR-TB management and treatment are contingent on early access to DSTs and intensive treatment [15]. Some studies concluded that diagnosis and treatment initiation delays are potentially associated with poor outcomes and low success rates [3,8]. The introduction of the GeneXpert method for DSTs may significantly speed up diagnosis [16] and MDR/RR-TB treatment initiation, consequently leading to improved outcomes [17]. However, different opinions implied that the implementation of GeneXpert, though lessening treatment delays, has not substantially contributed to reducing the treatment gap [18]. Moreover, the causes are still unclear for the prolonged waiting window between seeking to receive a DST from a medical provider and receiving the DST report to initiate effective treatment. In addition, the definition of delay varies across different studies, which hindered our understanding and comparison. For example, the diagnostic delay was defined as the period between the time when the sputum smear results were available and the time when the DST results were available in Zhang’s paper [7], while it was defined as the time interval from DST eligibility to DST results in Xu’s paper [3]. Last but not least, due to the difference between traditional DST and molecular biology’s test times, the impact on the analysis is also relatively large. Therefore, it is necessary and important to define the waiting time for DST results more accurately to avoid the impact of the test method. By addressing the causes of the delay and other associated factors, it can provide useful suggestions for DTP management to target solving problems such as poor access and high attrition rates and ultimately achieve higher success rates in MDR/RR-TB treatment. 

Therefore, the primary objective of this study was to explore patients’ waiting window from seeking a DST to receiving the DST report for treatment initiation. The study also discussed the factors associated with the waiting process, pre-treatment attrition, and treatment outcome. 

## 2. Materials and Methods

### 2.1. Study Setting 

**Figure 1:**
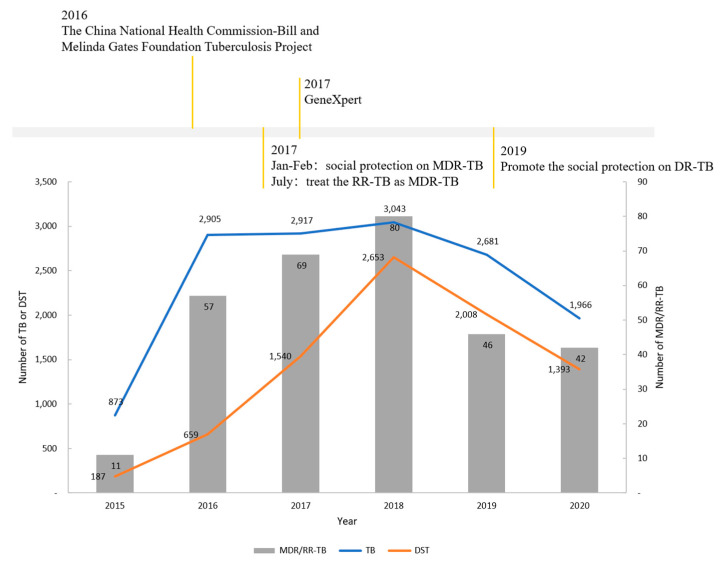
Number of patients diagnosed with MDR/RR-TB (grey), TB (blue), and number of people receiving DST (orange) on a yearly time scale from 1 September 2015 to 30 September 2020.

A retrospective cohort study based on record review was conducted in Taizhou, Zhejiang province, with a total of 6.62 million permanent residents across 3 districts, 3 county-level cities and 3 counties. The reported incidence of TB in Taizhou is 36.1/100,000 in 2021, with a male-to-female ratio of 2.54:1, and the patients were mainly farmers, workers, or unemployed [19]. From 2015 to 2020, the number of people registered with TB rose steadily and peaked in 2018, followed by a gradual decline that leveled the average number of reported cases to around 2500 per year. Since 2010, the modality of TB diagnosis and treatment management in Taizhou has gradually changed to become the “three-in-one” management mode, which involves the Centers for Disease Control (CDC), hospitals, and communities, with the integrated management of the CDC. Due to the constant practice of standardizing TB diagnosis and treatment management, the diagnosis and treatment levels were enhanced. A DST was conducted on five high-risk groups of TB patients, including (1) chronic TB patients (patients who experienced multiple treatment courses but remained sputum positive) and TB patients for whom retreatment failed; (2) close contacts of MDR-TB cases who developed sputum smear-positive (SS+) TB; (3) TB patients for whom initial treatment failed; (4) relapsed and returned TB patients; and (5) patients who remained smear-positive at the end of the third month of the initial treatment regimen [3]. After Nov. 2017, all new bacteriologically confirmed patients have also been advised to receive a DST. At the beginning of 2020, the eligibility was revised for all the patients mentioned above with bacteriologically confirmed. MDR-TB patients should be provided with a recommended regimen containing at least four effective second-line anti-TB drugs in a 6-month intensive phase and at least three drugs in an 18-month consolidation phase or a standardized shorter MDR-TB regimen according to the national MDR-TB prevention and control program [20]. In 2016, The China National Health Commission-Bill and Melinda Gates Foundation Tuberculosis Project (III) was initiated in Zhejiang [21], introducing GeneXpert to the medical facilities of several districts and counties in Taizhou. By late 2017, measurable improvements were seen on many levels, including the enhanced capability of MDR/RR-TB diagnosis, the widened coverage of drug-resistance screening, and expedited processes for clinical diagnosis and the treatment of MDR/RR-TB [13]. Meanwhile, several social protection policies were developed to improve patient access to TB care and treatment adherence (Figure 1). The number of patients receiving drug susceptibility tests (DSTs) increased sharply over three years, from less than 1000 before 2017 to 2653 patients in 2018. The number of MDR/RR-TB patients displayed the same trend as the number of patients receiving DSTs; patients diagnosed with MDR/RR-TB increased from 11 in Q4 of 2015 to 80 in 2018, and an approximation of 50 MDR/RR-TB cases per year (Figure 1). 

### 2.2. Data Collection and Definition

The data were collected from three national registration databases: TB register database (where TB-ID was used to identify a TB patient), the presumptive-MDR/RR-TB register database (where MDR/RR-TB patients are identified by TB-ID and MDR/RR-TB-ID), and the MDR/RR-TB register database (where MDR/RR-TB-ID was used to identify an MDR/RR-TB patient) in the CDC between 1 September 2015 and 30 September 2020. 

**Figure 2:**
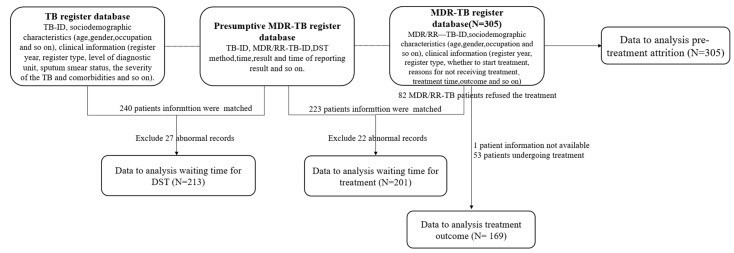
Flow diagram of data collection.

The study subjects were identified according to the MDR/RR-TB database. From the TB patient registration number (TB-ID) and MDR-TB patient registration number (MDR/RR-TB-ID), relevant types of data were extracted from the three databases, including basic information (age, gender, occupation, household register, distance to the designated hospital) and clinical information (register year, register type, level of diagnostic unit, sputum smear status at TB diagnosis, DST methods, the severity of the TB, and comorbidities). In addition, the dates of initial seeking medical care were extracted from the TB register database, the dates of receiving DST screening and DST results were collected from the presumptive-MDR/RR-TB register database, and the dates of treatment initiation and outcomes were also extracted from the MDR/RR-TB register database (Figure 2).

The definitions used in this study are as follows:

Waiting time for DST: the time between seeking medical care to visiting a designated diagnostic center for drug-susceptibility testing, and the time longer than the median waiting time for a DST was considered ‘long’ according to the relevant literature [8]. 

Pre-treatment attrition: was identified as patients without records of dates of treatment initiation. In a word, patients who did not initiate treatment were considered to be pre-treatment attrition. 

**Figure 3:**
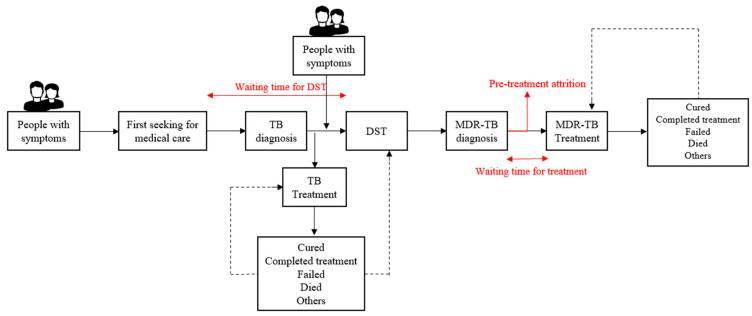
The diagnosis and treatment pathway for MDR/RR-TB patients and definitions used in the study.

Waiting time for treatment: the time between the date of diagnosis (date of reporting DST result) and the date of treatment initiation, and the waiting time longer than the median waiting time for treatment was considered to be ‘long’ according to the relevant literature [8] (Figure 3). 

Treatment outcomes were in accordance with WHO definitions [22]. Treatment success referred to ‘cured’ or ‘complete treatment’, whereas poor outcomes referred to ‘fail the treatment’, ‘died’, ‘lost to follow-up’, and ‘not evaluated’.

### 2.3. Statistical Analysis

R version 4.0.2 was used for analysis. Socio-demographic and clinical characteristics were summarized using means and standard deviations for continuous variables and counts/percentages for categorical variables. Univariate and multi-variates logistic regression analyses were used to explore the associated factors for waiting time for a DST, pre-treatment attrition, waiting time for treatment, and treatment outcome. In all of the multivariable analyses, variables were included on the basis of unadjusted *p* < 0.1, along with the authors’ prior knowledge. All of the statistical tests were two-tailed, and the *p*-value <0.1 and <0.05 were considered to indicate statistical significance in the univariate and multi-variate analysis, respectively.

## 3. Results

A total of 305 MDR/RR-TB patients were included in our analysis. Their mean age was 51.2 ± 17.5 years, 218 (71.5%) were males, and more than half of the patients were farmers or fishermen. Nearly one-third (86, 28.2%) of the patients were non-residents (immigrants for work or any other reasons and did not register in local public security bureaus), about half (168, 55.1%) of the patients were diagnosed with MDR/RR-TB after 2018 and registered in the CDC, over half (172, 56.4%) of the drug-resistant test were performed using the traditional method (Table 1).

### 3.1. Factors Associated with Waiting Time for DST

Of the 305 MDR/RR-TB patients, 240 had registration records of common TB. After excluding abnormal data, 213 were included in the analysis of the time of the drug susceptibility test. The median (IQR) time of patients from first visiting a hospital to receiving a drug susceptibility test was 24.00 (7.00, 66.00) days, and a time of >24 days was considered long. In the unadjusted analysis, the factors associated with long waiting times for a DST were the distance to the hospital (moderate), register year (before 2018), and severity (yes). In the adjusted analysis, non-residential patients were more likely to postpone the drug susceptibility test when compared with local patients (aOR: 2.89, 95% CI: 1.14–7.70). Patients registered before 2018 had nearly 19 times higher risk of not receiving a test when compared with patients registered after 2018 (aOR: 19.93, 95% CI: 8.99–48.51). Patients who first visited a county-level hospital experienced longer DST waiting times than patients who first visited a municipal hospital (aOR: 4.65, 95% CI: 1.08–21.67). Patients with negative baseline sputum smears were more likely not to receive a DST when compared with patients with a positive sputum smear (aOR: 3.54, 95% CI: 1.28–10.16). Patients reporting a history of comorbid pneumoconiosis had longer DST delays than patients with no comorbidities (aOR: 7.10, 95% CI: 1.23–47.98) (Table 2).

### 3.2. Factors Associated with Pre-Treatment Attrition

There were 82 MDR/RR-TB patients who refused the MDR/RR-TB treatment regimen, and the pre-treatment attrition was 26.9%. Gender, occupation, residency status, distance to the hospital, register year, patient type, drug-susceptibility test, and comorbidity were significantly associated with pre-treatment attrition. After adjusted analysis, elderly patients (≥60) when compared with ≤44 (aOR: 2.67, 95% CI: 1.16–6.35), non-residential patients when compared with local patients (aOR: 3.10, 95% CI: 1.55–6.30), patients registered before 2018 when compared with after 2018 (aOR: 2.17, 95% CI: 1.19–4.00) were more likely to not initiate MDR/RR treatment. However, housekeeping/unemployment and farmer/fisherman when compared with workers (aOR: 0.31, 95% CI: 0.10–0.92) and recurrent patients when compared with new cases (aOR: 0.43, 95% CI: 0.22–0.84) tended to pursue therapeutic measures actively (Table 3).

### 3.3. Factors Associated with Time of Waiting for Treatment

After excluding the abnormal data, 201 patients were included in the analysis. The median (IQR) waiting time for treatment was 30.00 (10.00, 87.00) days, and the waiting time >30 days was recognized as long. From the unadjusted analysis, occupation and the level of the diagnostic unit were associated with the waiting time for treatment. From the adjusted analysis, the patients who first visited a county-level hospital experienced longer waiting time than patients who visited a municipal hospital at first (aOR: 4.53, 95% CI: 1.19–22.59). Patients with a negative baseline sputum smear may spend more time waiting for treatment than patients with a positive sputum smear (aOR: 2.66, 95% CI: 1.12–6.59) (Table 4).

### 3.4. Treatment Outcome and Associated Factors

**Figure 4:**
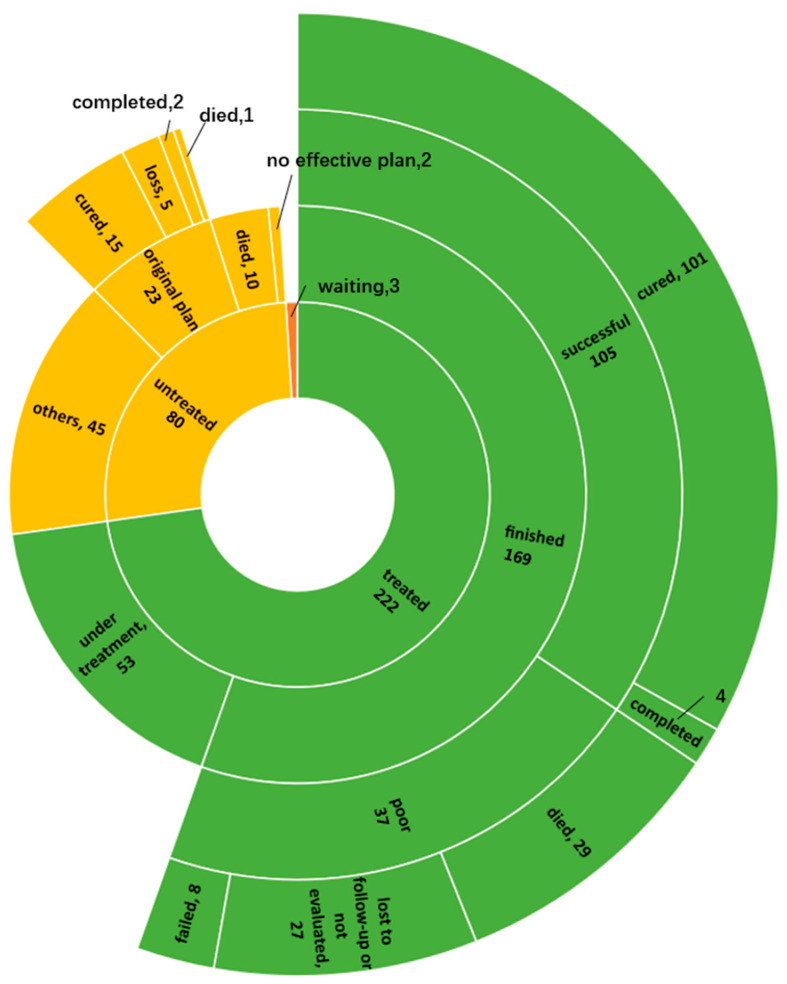
Outcome of 305 MDR/RR-TB patients.

Of the patients who did not initiate MDR/RR-TB treatment, 23 patients remained on the original therapeutic regimen with first-line drugs for drug-sensitive TB, and 17 patients achieved favorable outcomes. Of the patients who received the MDR/RR-TB treatment, 53 patients were still being treated, while 169 patients achieved a treatment outcome; of them, 105 patients achieved a favorable outcome, and the treatment success rate was 62.1% (Figure 4).

Regarding the factors associated with treatment outcome, age, occupation, register year, baseline sputum smear, and comorbidity may be related, as seen in the univariate logistic analysis. Moreover, elderly patients (≥60 years old) compared with ≤44 years old (aOR: 5.18, 95% CI: 1.53–19.98), patients with diabetes compared with no comorbidities (aOR: 5.48, 95% CI: 1.21–28.23), and the time for sputum smear conversion >1 month (aOR: 3.50, 95% CI: 1.36–9.70) had a high probability of poor prognosis in the multi-variate logistic analysis (Table 5).

## 4. Discussion

### 4.1. Waiting Time for DST was Still Long

In this study, we assessed the waiting time between receiving a DST and initiating treatment and the factors associated with the waiting time for a DST, treatment, pre-treatment attrition, and treatment outcomes. Considering that the clear evidence from other studies has found that efficient diagnostics tests can expedite the process of drug-resistance testing [23], the DST waiting time in our study excluded the duration between performing a DST and reporting DST results so as to avoid the impact of the detection time of DST methods, which is different from the diagnosis delay described by other published studies [3,7]. As a result, the median time from first seeking medical care to receiving a DST was 24 days. Through primary analysis, the reasons behind the DST delay are found to be both subjective and objective. The conventional DST method used to determine the susceptibility of isolates is based on a positive sputum culture, which could take 4–6 weeks [24]; however, it was replaced by more rapid molecular detection technology at the end of 2017. Thus, a registration year prior to 2018 was identified as the independent risk factor for an untimely DST; moreover, the social protections in Taizhou were also implemented around 2018. Migrant patients are more mobile, so it is more difficult to track and recall them for a DST [25]. Unlike prefecture-level medical institutions, county-level medical institutions need to transport the sputum specimen of presumptive MDR/RR-TB patients to qualified facilities to perform a DST, which extends the waiting timeframe [3]. Maybe the process of sample delivery and the feedback of the DST results would be smoother and take less time if the county-level medical institutions and prefectural designated MDR-TB hospitals had better coordination in place and were equipped with more necessary devices and trained technicians. Moreover, strengthening the DST capacity of county-level medical institutions may be another effective solution. Regarding the negative baseline sputum smear, it may result in taking more time to diagnose TB through a sputum culture or any other diagnosis technology, which could lead to an untimely DST [26]. The drug resistance of mycobacterium tuberculosis in patients with pneumoconiosis is very serious and difficult to cure [27]. Our study showed that MDR/RR-TB patients with pneumoconiosis were more susceptible to receiving an untimely DST partially due to the fact that the positive rates of the sputum smears of these patients were quite low [28]. It highlights the fact that TB patients with pneumoconiosis have a higher need for urgent attention and more on-time screening for drug resistance.

### 4.2. Pre-Treatment Attrition was Still High

Among 305 patients, pre-treatment attrition was 26.9%. The result is similar to other studies conducted in India and China [3], though relatively higher than the global figure (14%) reported in 2020 [2]. In our study, 34.1% of the patients were aged ≥60, and it has been assessed in other studies that patients ≥60 have less knowledge of medicine and health and low levels of cooperation, which both contribute to higher rates of treatment refusal in the elderly [29]. Nearly 70 percent of patients were housekeeping/unemployed people or farmers/fishermen. Their schedule is generally more flexible compared to the routine of workers, and they are probably not breadwinners, which drives the differences in adherence to treatment in terms of employment type. As discussed earlier, tracking and recalling workers for treatment is more difficult [25]. Social protections and the application of new technologies had not widely taken effect until 2018; on top of the financial burden [30], the OR of pre-treatment attrition before 2018 was two times more than post-2018. Similar to Caihong Xu’s study [3], the pre-treatment attrition rate of recurrent patients’ is lower than our results; supposedly, recurrent patients have a better understanding of TB and the importance of treatment. Therefore, it is necessary to strengthen the health education of the elderly and new TB patients. At the same time, we should pay attention to the social protections of workers, provide them with the necessary social subsidies, and strengthen management and follow-up to ensure that they can be treated in time.

### 4.3. Treatment Process Needs to Be Optimized

Regarding the waiting time for treatment, the median duration was 30 days, which is shorter than the same duration in India [8,31]; however, it still fails to meet the time required for timely treatment. The county-level medical institution and the negative sputum smear are two factors contributing to longer waiting times, as noted previously. A few approaches for reducing waiting times can be undertaken through the decentralization of MDR/RR-TB treatment instead of concentrating it in designated hospitals and the expansion of ambulatory models in healthcare systems [2,8].

The treatment success rate was 62.1%, similar to the results in other countries [32,33]. Although several studies have concluded that treatment delays were associated with poor treatment outcomes [8,9], our research reached a different conclusion. It could be explained by the different cut-off points of the short/long waiting time for treatment in different studies. Elderly people who disregard the importance of MDR/RR-TB treatment tend to have poor medication adherence and health condition [34], which is linked to the high possibility of poor treatment outcomes. Patients with diabetes are more likely to experience gastrointestinal problems, which can produce either delayed anti-TB drug absorption or malabsorption, ultimately resulting in poor treatment outcomes [35]. Similar to Lv’s study, shorter times for sputum conversion were associated with better treatment outcomes, which could provide a potential means for predicting MDR-TB treatment outcomes at an earlier stage [36]. Early attention should be paid, and the treatment regimen could be adjusted in time when the patient’s time for sputum conversion was too long. Meanwhile, society and healthcare agencies need to tend to patients who are ≥60 or have concomitant diabetes throughout the TB diagnosis and treatment process, clean the barriers from their treatment, and encourage them to adhere to the treatment.

### 4.4. Limitations

To the author’s best knowledge, the current study is one of studies that have analyzed DTP waiting times and explored the relevant factors and treatment outcomes based on MDR/RR-TB registration and management system. There are still some limitations in our study. First, due to the limited structure of the secondary data collected from the national databases, some sociodemographic and clinical predictors, such as education, income, smoking, alcohol, cavitation, and drug-use plan, may potentially affect waiting times or treatment outcomes, were not included in our survey. Second, our findings were not representative of the whole of Zhejiang province, as some patients were not included in our analysis due to limited access to the patient information needed for the analysis. Third, there is a likelihood of data errors given that the raw data extracted from the national registration databases were initially uploaded manually, despite the data verification and screening process we did prior to analysis. Moreover, our study ended on 30 September, 2020; Fei Huang’s study [37] suggested that the DTP of tuberculosis patients may have been affected by the COVID-19 epidemic during this period; for example, decreased laboratory-confirmed cases, but we did not specifically investigate and study this in this paper. Therefore, future studies should aim to cover a wider research area, obtain a larger sample size, collect more detailed sociodemographic and clinical information, and pay more attention to the impact of COVID-19 on the DTP of MDR/RR-TB patients.

## 5. Conclusions

In this study, we assessed several gaps in the MDR/RR-TB diagnosis and treatment process. We found that residency status, registration year, access to a prefecture-level hospital, diagnosis method, negative sputum smear, and comorbidity are factors that affect the waiting time at different levels. Patients ≥60 years old or with diabetes and a sputum smear conversion time of >1 month may lead to worse treatment outcomes. There is a need for immediate action that targets helping patients that fit the profile. More attention should be paid to the elderly or patients with comorbidities (especially pneumonoconiosis and diabetes) during the treatment. Improving the social protections as a means to encourage patients to cooperate with the treatment actively is a direct measure to take to shorten the turn-around time during the diagnosis and treatment process. Decentralizing MDR/RR-TB services, such as drug-resistance tests and MDR/RR-TB treatment, expanding the health workforce with trained health personnel and necessary medical equipment to smooth the DTP, providing a wider range of health education and consulting for the elderly or new TB patients are the same of importance.

## Figures and Tables

**Table 1:** Clinical and demographic characteristics of MDR/RR-TB patients. (N = 305).

Variable		No.	%
Age(years)	≤44	101	33.1
	45~59	100	32.8
	≥60	104	34.1
Gender	Male	218	71.5
	Female	87	28.5
Occupation	Worker	38	12.5
	Housekeeping or unemployment	53	17.4
	Farmer/fisherman	182	59.7
	Others	32	10.5
Residency status	Residents	219	71.8
	Non-residents	86	28.2
Distance to the hospital	Near	96	31.5
	Moderate	107	35.1
	Remote	102	33.4
Register year	After 2018	168	55.1
	Before 2018	137	44.9
Patient types	New case	169	55.4
	Recurrent	109	35.7
	Others	27	8.9
Level of diagnostic unit	Municipal	18	5.9
	County-level	222	72.8
	NA	65	21.3
Baseline sputum smear	Positive	251	82.3
	Negative	54	17.7
Drug-resistant test	Traditional	172	56.4
	Xpert	133	43.6
Drug susceptibility	MDR	216	70.8
	RR	76	24.9
	Others	13	4.3
Severity	No	195	63.9
	Yes	45	14.8
	NA	65	21.3
Comorbidity	No	183	60.0
	Pneumonoconiosis	11	3.6
	Diabetes	29	9.5
	Others	17	5.6
	NA	65	21.3

**Table 2:** Association of clinical and socio-demographic factors with waiting time for drug-susceptibility testing among MDR/RR-TB patients (N = 213).

	Waiting Time (Median [IQR])	Long Waiting Time ^a^ (N (%))	cOR (90%CI)	aOR (95%CI)
Age (years)				
≤44	23.00 [6.00, 54.00]	39 (48.1)	1.00	1.00
45~59	38.00 [8.00, 82.75]	33 (51.6)	1.15 (0.66–1.99)	0.65 (0.25–1.69)
≥60	26.00 [9.25, 50.25]	34 (50.0)	1.08 (0.63–1.85)	1.68 (0.63–4.60)
Gender				
Male	23.50 [8.00, 65.25]	74 (49.3)	1.00	1.00
Female	28.00 [6.50, 66.50]	32 (50.8)	1.06 (0.65–1.74)	0.9 (0.41–1.95)
Occupation				
Worker	29.00 [11.00, 55.00]	18 (54.5)	1.00	1.00
Housekeeping/ unemployment	14.00 [8.50, 48.00]	14 (35.9)	0.47 (0.21–1.03)	0.7 (0.18–2.81)
Farmer/fisherman	28.00 [5.75, 66.25]	61 (52.6)	0.92 (0.48–1.77)	2 (0.58–7.44)
Others	28.00 [10.00, 166.00]	13 (52.0)	0.9 (0.38–2.17)	1.3 (0.28–6.23)
Residency status				
Residents	23.00 [7.00, 72.25]	78 (48.1)	1.00	1.00
Non-residents	29.00 [8.00, 54.00]	28 (54.9)	1.31 (0.77–2.24)	2.89 (1.14–7.70) **
Distance to the hospital				
Near	35.00 [12.00, 73.00]	39 (60.0)	1.00	1.00
Moderate	14.00 [4.00, 51.50]	33 (41.8)	0.48 (0.27–0.83) **	0.44 (0.16–1.14)
Remote	24.00 [7.00, 62.00]	34 (49.3)	0.65 (0.36–1.15)	0.46 (0.16–1.25)
Register year				
After 2018	10.00 [3.00, 24.50]	28 (25.2)	1.00	1.00
Before 2018	50.00 [28.00, 134.75]	78 (76.5)	9.63 (5.76–16.51) *	19.93 (8.99–48.51) **
Patient types				
New case	24.00 [6.00, 62.50]	61 (49.6)	1.00	1.00
Recurrent	19.00 [9.00, 66.00]	33 (45.2)	0.84 (0.51–1.36)	0.67 (0.32–1.40)
Others	41.00 [14.00, 135.00]	12 (70.6)	2.44 (1.00–6.54)	1.10 (0.25–5.17)
Level of diagnostic unit				
Municipal	10.00 [2.75, 34.75]	6 (37.5)	1.00	1.00
County-level	25.00 [7.00, 73.00]	100 (50.8)	1.72 (0.72–4.32)	4.65 (1.08–21.67) **
Baseline sputum smear				
Positive	24.00 [7.00, 68.00]	90 (49.2)	1.00	1.00
Negative	27.50 [7.00, 41.00]	16 (53.3)	1.18 (0.62–2.28)	3.54 (1.28–10.16) **
Severity				
No	21.00 [5.75, 57.25]	79 (45.9)	1.00	1.00
Yes	42.00 [13.00, 77.00]	27 (65.9)	2.27 (1.26–4.19) **	1.29 (0.46–3.68)
Comorbidity				
No	24.00 [7.00, 66.50]	80 (49.1)	1.00	1.00
Pneumonoconiosis	36.00 [12.00, 50.00]	6 (66.7)	2.07 (0.66–7.60)	7.10 (1.23–47.98) **
Diabetes	15.00 [10.00, 56.00]	11 (44.0)	0.82 (0.40–1.66)	0.98 (0.30–3.12)
Others	31.50 [4.75, 77.00]	9 (56.2)	1.33 (0.56–3.25)	0.30 (0.08–1.16)

^a^ The time longer than the median of waiting time for DST. * *p* < 0.1 ** *p* < 0.05.

**Table 3:** Association of clinical and socio-demographic factors with pre-treatment attrition among MDR/RR-TB patients (N = 305).

Variable	No (N (%))	Yes (N (%))	cOR (90%CI)	aOR (95%CI)
Age (years)				
≤44	70 (69.3)	31 (30.7)	1.00	1.00
45~59	74 (74.0)	26 (26.0)	0.79 (0.47–1.33)	1.84 (0.84–4.11)
≥60	79 (76.0)	25 (24.0)	0.71 (0.42–1.20)	2.67 (1.16–6.35) **
Gender				
Male	167 (76.6)	51 (23.4)	1.00	1.00
Female	56 (64.4)	31 (35.6)	1.81 (1.15–2.85) **	1.70 (0.92–3.13)
Occupation				
Worker	17 (44.7)	21 (55.3)	1.00	1.00
Housekeeping/unemployment	39 (73.6)	14 (26.4)	0.29 (0.14–0.60) **	0.31 (0.10–0.92) **
Farmer/fisherman	147 (80.8)	35 (19.2)	0.19 (0.10–0.36) **	0.20 (0.08–0.53) **
Others	20 (62.5)	12 (37.5)	0.49 (0.21–1.08)	0.48 (0.15–1.50)
Residency status				
Residents	172 (78.5)	47 (21.5)	1.00	1.00
Non-residents	51 (59.3)	35 (40.7)	2.51 (1.60–3.95) **	3.10 (1.55–6.30) **
Distance to the hospital				
Near	64 (66.7)	32 (33.3)	1.00	1.00
Moderate	79 (73.8)	28 (26.2)	0.71 (0.43–1.18)	0.72 (0.35–1.48)
Remote	80 (78.4)	22 (21.6)	0.55 (0.32–0.93) *	0.68 (0.33–1.40)
Register year				
After 2018	133 (79.2)	35 (20.8)	1.00	1.00
Before 2018	90 (65.7)	47 (34.3)	1.98 (1.29–3.06) **	2.17 (1.19–4.00) **
Patient types				
New case	118 (69.8)	51 (30.2)	1.00	1.00
Recurrent	90 (82.6)	19 (17.4)	0.49 (0.29–0.80) **	0.43 (0.22–0.84) **
Others	15 (55.6)	12 (44.4)	1.85 (0.92–3.70)	1.15 (0.40–3.22)
Level of diagnostic unit				
Municipal	12 (66.7)	6 (33.3)	1.00	
County-level	157 (70.7)	65 (29.3)	0.83 (0.36–2.04)	-
NA	54 (83.1)	11 (16.9)	0.41 (0.15–1.12)	-
Baseline sputum smear				
Positive	181 (72.1)	70 (27.9)	1.00	
Negative	42 (77.8)	12 (22.2)	0.74 (0.40–1.30)	-
Drug-resistant test				
Traditional	112 (65.1)	60 (34.9)	1.00	
Xpert	111 (83.5)	22 (16.5)	0.37 (0.23–0.58) $	-
Drug susceptibility				
MDR	160 (74,1)	56 (25.9)	1.00	
RR	51 (67.1)	25 (32.9)	1.4 (0.86–2.25)	-
Others	12 (92.3)	1 (7.7)	0.24 (0.02–1.00)	-
Severity				
No	141 (72.3)	54 (27.7)	1.00	
Yes	28 (62.2)	17 (37.8)	1.59 (0.89–2.79)	-
NA	54 (83.1)	11 (16.9)	0.53 (0.28–0.95) $	-
Comorbidity				
No	122 (66.7)	61 (33.3)	1.00	1.00
Pneumonoconiosis	11 (100.0)	0 (0.0)	0 (NA-infinite)	0 (NA-infinite)
Diabetes	24 (82.8)	5 (17.2)	0.42 (0.16–0.92) *	0.49 (0.14–1.46)
Others	12 (70.6)	5 (29.4)	0.83 (0.31–2.00)	1.10 (0.31–3.49)
NA	54 (83.1)	11 (16.9)	0.41 (0.22–0.73) **	0.33 (0.13–0.74)

* *p* < 0.1; ** *p* < 0.05. $ The variable would not be included in the multi logistic regression.

**Table 4:** Association of clinical and socio-demographic factors with waiting time for treatment among MDR/RR-TB patients (N = 201).

Variable	Waiting Time (Median [IQR])	Long (N (%))	cOR (90%CI)	aOR (95%CI)
Age (years)				
≤44	22.00 [9.50, 55.50]	27 (42.9)	1.00	1.00
45~59	30.50 [12.25, 94.00]	33 (50.0)	1.33 (0.75–2.40)	1.46 (0.63–3.41)
≥60	34.00 [8.00, 104.50]	37 (51.4)	1.41 (0.80–2.50)	1.7 (0.74–3.94)
Gender				
Male	33.00 [10.00, 98.00]	78 (52.3)	1.00	1.00
Female	20.50 [9.75, 57.75]	19 (36.5)	0.52 (0.30–0.90)	0.52 (0.25–1.05)
Occupation				
Worker	55.00 [30.00, 123.00]	12 (70.6)	1.00	1.00
Housekeeping/unemployment	107.50 [21.00, 188.50]	23 (67.6)	0.87 (0.29–2.48)	0.65 (0.15–2.55)
Farmer/fisherman	23.00 [9.00, 76.00]	58 (43.6)	0.32 (0.12–0.78) **	0.37 (0.10–1.23)
Others	17.00 [6.00, 24.00]	4 (23.5)	0.13 (0.03–0.44) **	0.11 (0.02–0.52)
Residency status				
Residents	32.00 [10.00, 88.00]	80 (51.3)	1.00	
Non-residents	18.00 [9.00, 82.00]	17 (37.8)	0.58 (0.32–1.01)	-
Distance to the hospital				
Near	18.00 [5.75, 70.75]	23 (41.1)	1.00	
Moderate	48.00 [8.00, 128.00]	39 (54.2)	1.7 (0.94–3.08)	-
Remote	30.00 [14.00, 82.00]	35 (47.9)	1.32 (0.73–2.39)	-
Register year				
After 2018	32.00 [10.00, 96.75]	63 (51.6)	1.00	
Before 2018	24.00 [8.50, 74.00]	34 (43.0)	0.71 (0.44–1.14)	-
Patient types				
New case	23.00 [8.00, 80.00]	48 (44.0)	1.00	
Recurrent	42.00 [13.00, 127.00]	45 (55.6)	1.59 (0.98–2.59)	-
Others	17.00 [7.00, 59.50]	4 (36.4)	0.73 (0.23–2.08)	-
Level of diagnostic unit				
Municipal	5.50 [2.00, 18.50]	3 (25.0)	1.00	1.00
County-level	42.00 [13.00, 98.00]	82 (56.6)	3.9 (1.35–13.72) **	4.53 (1.19–22.59) **
NA	14.50 [7.50, 48.25]	12 (27.3)	1.12 (0.35–4.27)	1.09 (0.25–5.82)
Baseline sputum smear				
Positive	27.00 [10.00, 87.00]	77 (46.7)	1.00	1.00
Negative	47.00 [8.00, 100.75]	20 (55.6)	1.43 (0.78–2.64)	2.66 (1.12–6.59) **
Drug-resistant test				
Traditional	31.00 [12.00, 95.00]	50 (51.5)	1.00	
Xpert	23.50 [9.00, 85.25]	47 (45.2)	0.78 (0.49–1.23)	-
Drug susceptibility				
MDR	31.00 [10.00, 87.00]	74 (51.0)	1.00	
RR	20.50 [9.25, 75.50]	19 (41.3)	0.68 (0.34–1.31)	-
Others	27.50 [23.25, 114.00]	4 (40.0)	0.64 (0.16–2.33)	-
Severity				
No	34.50 [11.00, 105.75]	72 (54.5)	1.00	
Yes	33.00 [17.00, 76.00]	13 (52.0)	0.9 (0.44–1.86)	-
NA	14.50 [7.50, 48.25]	12 (27.3)	0.31 (0.16–0.58) $	-
Comorbidity				
No	31.00 [9.00, 97.00]	58 (51.3)	1.00	
Pneumonoconiosis	111.00 [31.50, 252.75]	7 (70.0)	2.21 (0.72–8.04)	-
Diabetes	61.00 [25.25, 93.00]	15 (68.2)	2.03 (0.92–4.76)	-
Others	26.50 [13.50, 46.00]	5 (41.7)	0.68 (0.24–1.85)	-
NA	14.50 [7.50, 48.25]	12 (27.3)	0.36 (0.18–0.66) $	-

* *p* < 0.1 ** *p* < 0.05. $ The variable would not be included in the multi logistic regression.

**Table 5:** Association of clinical and socio-demographic factors with treatment outcome among MDR/RR-TB patients (N = 142).

Variable	Successful (N(%))	Poor (N(%))	cOR (90%CI)	aOR (95%CI)
Age (years)				
≤44	43 (76.8)	13 (23.2)	1.00	1.00
45~59	34 (64.2)	19 (35.8)	1.85 (0.92–3.77)	2.02 (0.57–7.73)
≥60	28 (46.7)	32 (53.3)	3.78 (1.96–7.54) **	5.18 (1.53–19.98) **
Gender				
Male	75 (60.0)	50 (40.0)	1.00	1.00
Female	30 (68.2)	14 (31.8)	0.70 (0.37–1.28)	0.40 (0.13–1.15)
Occupation				
Worker	13 (81.2)	3 (18.8)	1.00	1.00
Housekeeping / unemployment	20 (62.5)	12 (37.5)	2.60 (0.82–9.78)	1.04 (0.15–8.00)
Farmer/fisherman	59 (55.7)	47 (44.3)	3.45 (1.25–11.89) *	0.78 (0.14–4.81)
Others	13 ((86.7)	2 (13.3)	0.67 (0.11–3.38)	0 (0-infinite)
Residency status				
Residents	84 (62.7)	50 (37.3)	1.00	
Non-residents	21 (60.0)	14 (40.0)	1.12 (0.58–2.11)	-
Distance to the hospital				
Near	30 (63.8)	17 (36.2)	1.00	
Moderate	41 (67.2)	20 (32.8)	0.86 (0.44–1.69)	-
Remote	34 (55.7)	27 (44.3)	1.40 (0.73–2.72)	-
Register year				
After 2018	59 (69.4)	26 (30.6)	1.00	1.00
Before 2018	46 (54.8)	38 (45.2)	1.87 (1.11–3.20) *	2.06 (0.81–5.35)
Patients types				
New case	59 (65.6)	31 (34.4)	1.00	
Recurrent	37 (55.2)	30 (44.8)	0.9 (0.47–1.81)	-
Others	9 (75.0)	3 (25.0)	1.17 (0.41–3.19)	-
Level of diagnostic unit				
Municipal	5 (50.0)	5 (50.0)	1.00	
County-level	78 (65.0)	42 (35.0)	0.54 (0.18–1.63)	-
NA	22 (56.4)	17 (43.6)	0.77 (0.24–2.53)	-
Baseline sputum smear				
Positive	83 (59.3)	57 (40.7)	1.00	1.00
Negative	22 (75.9)	7 (24.1)	0.46 (0.21–0.97) *	0.76 (0.15–3.31)
Drug-resistant test				
Traditional	57 (57.6)	42 (42.4)	1.00	
Xpert	48 (68.6)	22 (31.4)	0.62 (0.36–1.06)	-
Drug susceptibility				
MDR	77 (73.2)	47 (37.9)	1.00	
RR	21 (77.8)	12 (36.4)	0.94 (0.47–1.81)	-
Others	7 (63.6)	5 (41.7)	1.17 (0.41–3.19)	-
Severity				
No	69 (65.1)	37 (34.9)	1.00	
Yes	14 (58.3)	10 (41.7)	1.33 (0.61–2.83)	-
NA	22 (56.4)	17 (43.6)	1.44 (0.77–2.70)	-
Comorbidity				
No	65 (69.9)	28 (30.1)	1.00	1.00
Pneumonoconiosis	6 (66.7)	3 (33.3)	1.16 (0.31–3.78)	0.81 (0.10–5.04)
Diabetes	8 (50.0)	8 (50.0)	2.32 (0.93–5.78)	5.48 (1.21–28.23) **
Others	4 (33.3)	8 (66.7)	4.64 (1.64–14.59) **	3.19 (0.61–18.81)
NA	22 (56.4)	17 (43.6)	1.79 (0.93–3.43)	2.90 (0.89–10.28)
Waiting for treatment #				
Short	42(56.8)	32 (43.2)	1.00	1.00
Long	46(62.2)	28 (37.8)	0.80 (0.46–1.39)	0.48 (0.18–1.21)
Time for sputum smear conversion §				
≤1 month	65(70.7)	27 (29.3)	1.00	1.00
>1 month	31(58.5)	22 (41.5)	1.71 (0.94–3.10)	3.50 (1.36–9.70) **

* *p* < 0.1 ** *p* < 0.05. # 21 patients deleted from the 169 observations since the abnormal waiting time for treatment. § 24 patients deleted from the 169 observations since the missing data for time of sputum smear turning negative.

## Data Availability

The data presented in this study are available upon request from the corresponding author.
